# T1 mapping: useful for treatment monitoring in patients with senile systemic amyloidosis?

**DOI:** 10.1186/1532-429X-16-S1-P294

**Published:** 2014-01-16

**Authors:** Fabian aus dem Siepen, Arnt V Kristen, Henning Steen, Florian Andre, Sebastian A Seitz, Evangelos Giannitsis, Grigorios Korosoglou, Hugo A Katus, Sebastian Buss

**Affiliations:** 1 Department of Cardiology, University of Heidelberg, Heidelberg, Germany

## Background

Recent reports indicate that epigallocatechin-3-gallate (EGCG), the most abundant catechin in green tea, is potent to inhibit fibril formation of several amyloidogenic proteins in vitro. In vivo studies revealed reduction of left ventricular myocardial mass (LVM) after 12 months of daily consumption of 450 mg EGCG. However, the underlying process of LVM reduction, either due to reduction of amyloid or due to atrophy of cardiomyocytes, remained unknown. T1-mapping has the potential to monitor the extent of the extracellular volume (ECV). We sought to investigate the use of T1 mapping for monitoring of treatment effects in a cohort of patients with senile systemic amyloidosis (SSA) treated with EGCG for 12 months.

## Methods

**Figure 1:**
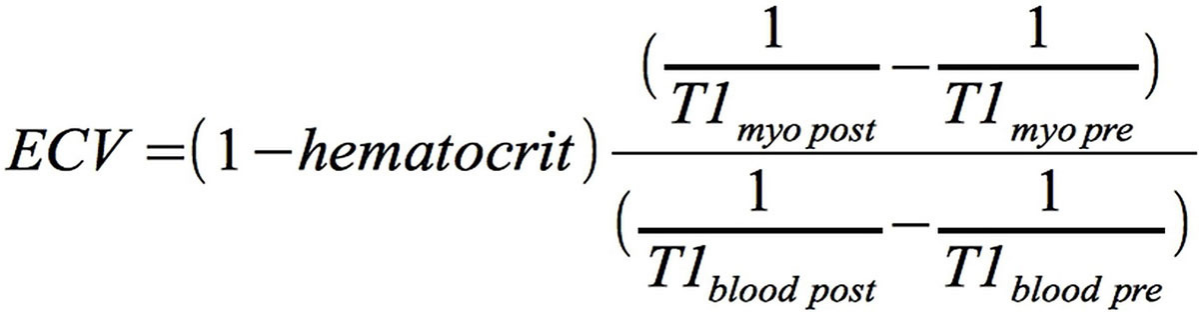
**T1 myo pre: native T1 relaxation time for myocardium T1 myo post: post-contrast T1 relaxation time for myocardium T1 blood pre: native T1 relaxation time for blood T1 blood post: post-contrast T1 relaxation time for blood**.

CMR examinations were performed in 8 patients (70 ± 8 years, 7 males) with histologically proven SSA before and 12 months after daily consumption of 450 mg EGCG using a 1.5 T CMR scanner (Achieva, Philips Healthcare). Short axis slices were acquired using SSFP-sequences to measure left ventricular volumes, ejection fraction (EF) and LVM. T1-maps were created out of 11 mid-ventricular short axis views with increasing inversion times (TI; 100-4400 msec) using a single breath-hold modified Look-Locker inversion-recovery sequence (MOLLI, TR/TE = 3,5/1,8 msec, flip angle = 35°) in late diastole before and 15 minutes after injection of gadolinium-DTPA contrast agent (0.2 mmol/kg body weight). ECV was calculated using the formula given in Figure 1.

## Results

**Figure 2:**
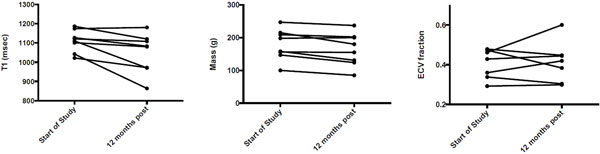


After 12 months of EGCG consumption a significant decrease of LVM (-14.5 ± 12.9 g, p < 0.05, Figure 2) was observed. Moreover, a significant decrease of native T1 (-63.3 ± 64.1 ms, p < 0.05, Figure 2) was noticed. There was no significant change in ECV and EF.

## Conclusions

This is a preliminary study to evaluate T1-mapping for the monitoring of treatment effects in patients with SSA. The decrease of T1 relaxation time after 12 months of EGCG treatment might possibly indicate a reduction of amyloid load. Thus, T1-mapping might be a potential tool for monitoring further experimental therapies. However, the considerable gap between native T1 and ECV measurements remain unexplained and needs to be investigated in a future study.

## Funding

None.

